# Advanced scanning probe lithography using anatase-to-rutile transition to create localized TiO_2_ nanorods

**DOI:** 10.3762/bjnano.10.40

**Published:** 2019-02-08

**Authors:** Julian Kalb, Vanessa Knittel, Lukas Schmidt-Mende

**Affiliations:** 1 Department of Physics, University of Konstanz, Universitätsstraße 10, 78457 Konstanz, Germany

**Keywords:** hydrothermal crystal growth, lithography, nanostructures, seed crystals, surface processes, oxides

## Abstract

In this article, we demonstrate the position-controlled hydrothermal growth of rutile TiO_2_ nanorods using a new scanning probe lithography method in which a silicon tip, commonly used for atomic force microscopy, was pulled across an anatase TiO_2_ film. This process scratches the film causing tiny anatase TiO_2_ nanoparticles to form on the surface. According to previous reports, these anatase particles convert into rutile nanocrystals and provide the growth of rutile TiO_2_ nanorods in well-defined areas. Due to the small tip radius, the resolution of this method is excellent and the method is quite inexpensive compared to electron-beam lithography and similar methods providing a position-controlled growth of semiconducting TiO_2_ nanostructures.

## Introduction

Rutile TiO_2_ is a chemically stable semiconductor with a band gap of 3.1 eV [1]. Dependent on the kind of nanostructure and doping, it has outstanding electronic and optoelectronic characteristics. The fabrication of nanostructured TiO_2_ is inexpensive and hence employed in many applications such as photodetectors [2], photovoltaics [3–6], photocatalysis [7–11], surficial disinfection [12], biosensing [13], gas sensing [14–16], dewetting [17–19], fuel cells [20], lithium batteries [21–26], field-emission devices [27], data storage devices [28], gaso- and electrochromic displays [29–30], and nonlinear optical devices [31]. Even in the field of medical engineering, such structures are promising candidates for improving the adhesion between implants and body tissues [32]. Many of the listed applications could be refined into spatially resolved technologies such as locally controlled photocatalysis for molecule degradation, spatially resolved gas or molecule sensing, gradients on superhydrophilic surfaces with close-meshed contrast, local light scattering, high-resolution surface roughness gradients or microchannels. This set of extensions for rutile TiO_2_ nanorod applications is a valuable toolkit for lab-on-a-chip devices [33–35].

**Figure 1:**
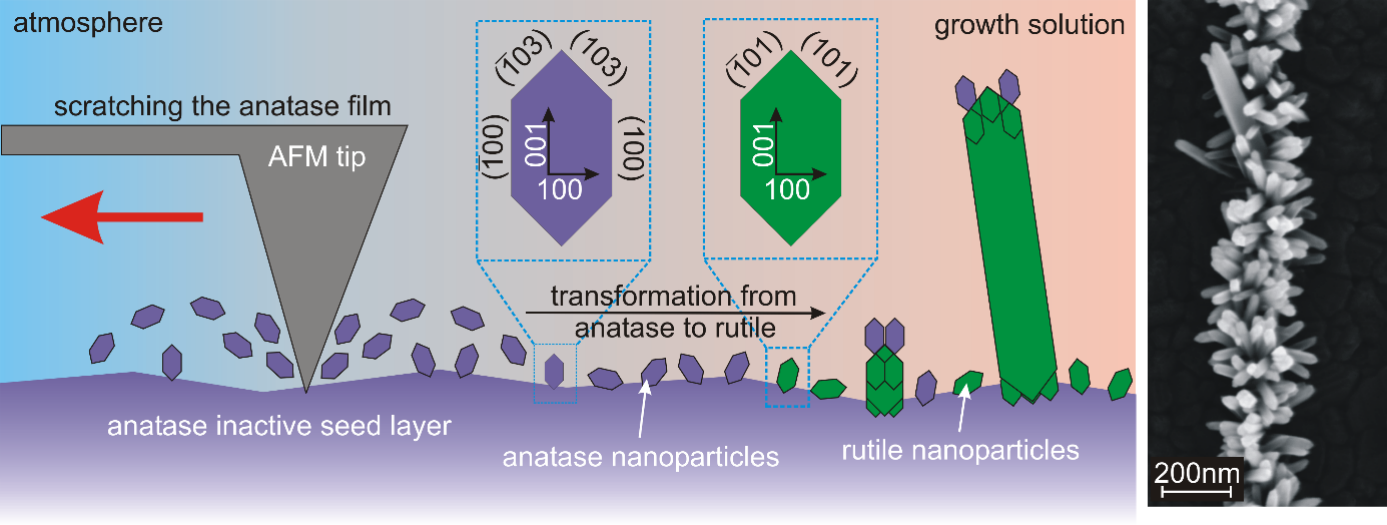
Schematic drawing of activating inactive anatase TiO_2_ films: Scratching with an AFM tip causes the formation of anatase nanoparticles. In the growth solution, these anatase particles are transformed into rutile particles and promote the growth of rutile TiO_2_ nanorods as described by Li et al. [37]. The SEM image displays the resulting row of nanorods obtained with the method presented in this report.

Recently, we investigated the influence of rutile seed layers on the growth and shape of hydrothermally grown rutile TiO_2_ nanorods [36]. Beside the homogeneous growth on macroscopic areas, we indicated how to trigger the growth via conventional electron-beam lithography locally. In this report, we apply an advanced but inexpensive scanning probe lithography technique to draw thin lines of nanorods directly on polycrystalline anatase TiO_2_ films. The resulting nanorod arrangements are compared with similar structures obtained with conventional electron-beam lithography, which is a more expensive and laborious procedure. The method is drafted in Figure 1. A silicon tip, as it is used in a conventional atomic force microscope (AFM), was pulled across an anatase film. During this process, surface defects are created as well as dust that contains tiny anatase TiO_2_ nanoparticles. Due to the lattice mismatch between anatase and rutile, in general, rutile nanorods do not grow on anatase crystal facets. The work of Li et al. and Sclafani et al. forms the basis of the development of the presented technique. They assume the transformation of anatase into rutile nanoparticles to be major process during the hydrothermal growth of rutile TiO_2_ nanorods [37–38]. It was observed by different groups that rutile TiO_2_ nanorods grow hydrothermally on anatase particles with a diameter of approximately 25 nm [39–40]. Even if rutile TiO_2_ seed nanocrystals are already present, it is assumed that anatase clusters diffuse along rutile TiO_2_ surfaces towards low-energy facets and perform a solid-state phase transformation into rutile TiO_2_ on these facets [41]. Thus, it is possible to promote a position-controlled hydrothermal growth by generating anatase nanoparticles locally by scratching across an anatase film using a conventional AFM tip.

## Experimental

We fabricated a 40 nm thin amorphous TiO_2_ film by DC sputter deposition on a polished (100) silicon wafer with a native thin SiO*_x_* layer. Sputter deposition was performed at room temperature with a Gamma 1000C sputter system (Surrey NanoSystems Ltd) and a TiO_2_ sputter target (99.99% purity, Testbourne Ltd). The deposition rate was set to 0.2 Å/s and the process was done in argon (20 sccm) at a pressure of 6.7 × 10^−3^ mbar. Subsequently, the sample was annealed at 850 °C in oxygen (500 sccm) for 2 h resulting in a polycrystalline anatase TiO_2_ film. Optionally, an additional 3 nm thin SiO_2_ layer was placed on the anatase film with RC sputter deposition. Scanning probe lithography was performed with an Innova AFM (Bruker) in contact mode. The applied force was significantly higher than usually chosen for topography scanning. We used OTESPA-R3 (Bruker AFM probes) silicon tips with a spring constant of approximately 26 N/m. Based on the spring constant, we approximated that the average applied force on the tip was 8 µN. The tip radius is less than 10 nm before scratching but it is enlarged during the lithography process due to abrasion. More details about the worn probes are found in Supporting Information File 1. Unless otherwise noted, each line was scratched for 256 times (128 times in each direction) in order to generate a sufficient amount of seeds. The technique was performed under ambient conditions with a writing speed of 10 μm/s (scan frequency: 0.5 Hz; line length: 10 μm). After seed generation, the samples were placed top-side down in a Teflon liner and autoclaved with a hydrothermal growth solution consisting of 20 mL hydrochloric acid (14.8 wt %, diluted in distilled water) and 350 μL titanium(IV) butoxide in an oven with a setpoint temperature of 180 °C for 75 min. The hydrothermal growth process was stopped by cooling down the autoclave in cold tap water. Scanning electron microscopy (SEM) images were taken with a Zeiss CrossBeam 1540XB. An Olympus BX51 optical microscope with a 250× objective was employed for bright-field optical images. For reference samples prepared by electron-beam lithography, poly(methyl methacrylate) (PMMA) 950 A2 was used as an electron resist and spin-coated at 3000 rpm for 90 s with a ramp of 1000 rpm/s. Subsequently, the resist was dried at 120 °C on a hotplate and prebaked at 170 °C in an oven for 30 min. The 30 nm thick resist layer was covered with an 8–10 nm thick aluminium film, which was deposited via resistive evaporation with a deposition rate of 3 Å*/*s. The Zeiss CrossBeam 1540XB FE SEM was employed for electron-beam exposure using an acceleration voltage of 10 kV, a current of 25 pA, a dose of 200 μAs/cm^2^, an area step size and dwell time of 1*.*6 nm and 200 ns, respectively, and an aperture of 10 μm. The aluminium was dissolved in a 0.5 molar NaOH bath within 12 s. The samples were developed in methyl isobutyl ketone (MIBK) for 25 s and cleaned with isopropanol. In case of electron-beam lithography, a polycrystalline rutile TiO_2_ films was fabricated as a seed layer for the hydrothermal growth process as we described in detail in our previous work [36]. For that purpose, a 40 nm thin titanium layer was deposited via electron-beam assisted evaporation from titanium pellets (99.995% purity, Kurt J. Lesker Ltd.) at a pressure of 1*.*5 × 10^−6^ mbar with a deposition rate of 0.9 Å/s. Titanium was turned into the TiO_2_ seed layer in a rapid thermal processing oven at 850 °C in an oxygen atmosphere (500 sccm) for 2 h using a temperature dwell time of ±1 °C/s. The remaining resist was dissolved in acetone.

## Results and Discussion

**Figure 2:**
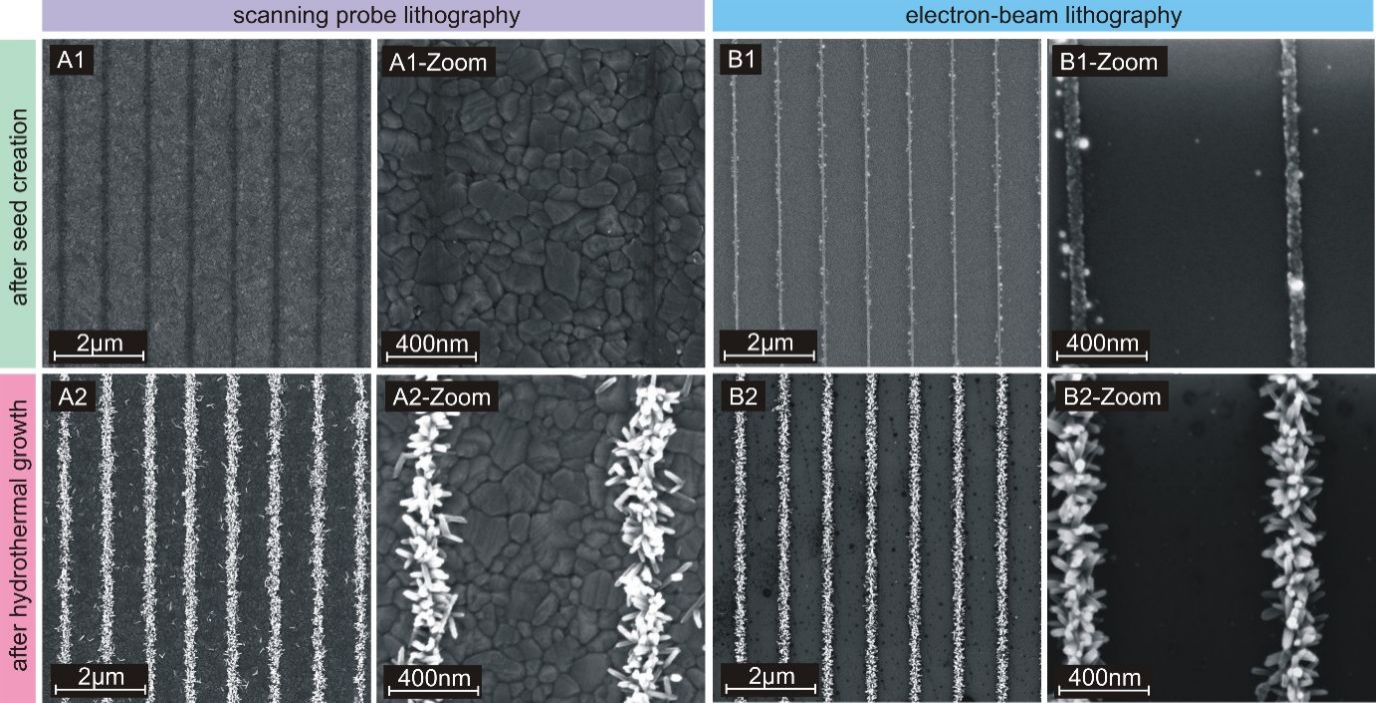
SEM images with different magnifications after scratching (panel A1, using scanning probe lithography) and seed fabrication (panel B1, using electron-beam lithography), and before hydrothermal growth; line array consisting of nanorods created with the hydrothermal growth process (panel A2 and panel B2 for scanning probe and electron-beam lithography, respectively). The stripe width is less than 100 nm, the pitch is 1 μm and the rod length is approximately 100 nm. B1- and B2-Zoom are reprinted with permission from our previous work [36], copyright 2018 Elsevier.

We have shown in a previous study that the nanorods produced by the described hydrothermal growth method have a rutile crystal structure [42]. The resulting nanostructures obtained with scanning probe lithography are presented in Figure 2 by SEM. For comparison, the same structures were fabricated with electron-beam lithography to demonstrate that both techniques yield equal results. In case of conventional electron-beam lithography, titanium was deposited on a patterned electron resist. After removing the mask, the titanium was oxidized at 850 °C resulting in polycrystalline rutile seeds along the pattern lines as we described previously [36]. The seed grains have diameters between 5 and 100 nm and hence, their dimensions are similar to those of the nanorods. Instead of using a mask, scanning probe lithography generates locally anatase nanoparticles by scraping an AFM tip across an anatase film. The transformation of anatase nanoparticles into rutile nanocrystals happens during the hydrothermal growth process at temperatures above 120 °C as described by Li et al. [37]. The resulting rutile seeds are significantly smaller than the final dimensions of the rutile nanorods.

### Excluding competing processes resulting in localized growth of nanorods

**Figure 3:**
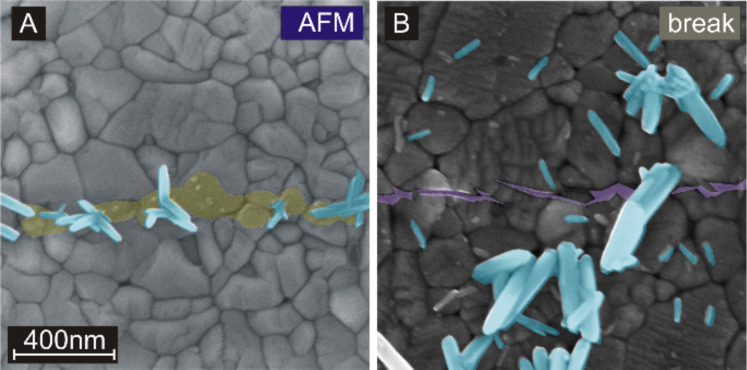
A) SEM image after short hydrothermal growth on a scratched line. The line is written only a few times in order to generate fewer growth sites compared to Figure 2. For clarity, nanorods are colored bluish and the scarified surface is colored yellowish. B) SEM image after applying several times the hydrothermal growth to a stress-induced line of breakage. For clarity, the breaking line is colored purple. The breaking line does not specifically promote the growth of nanorods. Nanorods growing around the breaking line might originate from anatase nanoparticles released during the breaking process.

Before specific properties of this technique are discussed, it should be excluded that AFM tip-induced growth is not simply a surface roughening effect. In Figure 3, the difference between a scratched film (panel A) and a mechanically broken film (panel B) is demonstrated. The scratched line in Figure 3A is marked yellowish, the breaking line in Figure 3B is marked purple. The breaking line was induced by mechanical stress generated by scratching the sample with a diamond writer manually. The mechanical stress was guided through the sample from the center of the scratch to surrounding regions. The film was removed close to the scratch, but tiny cracks such as the one shown in the figure appear in a distance of a few micrometers. The fracture continues in grains or grain boundaries indicating preferred breaking lines along specific crystal planes. Nanorods grow only in the scratched area, while there is no preferred growth observable at the breaking line. Nanorods growing around the breaking line might originate from anatase nanoparticles released during the breaking process.

Besides surface roughening, charging is another candidate that could promote the growth on scratched regions. The tip causes a lot of friction and might charge the film locally. The charges are likely trapped in defect states generated by the scratching process. Furthermore, the native silicon oxide layer prevents a quick charge transport into the conductive boron-doped silicon substrate. A charged film might attract the precursor more likely and the density of rods would be increased. However, nanorods grown on anatase films resist even long sonication, which is more reasonable for nanorods being linked to nanoparticles bound to the anatase film rather than nanorods bound to the seed layer by Coulomb interaction only.

### Special features of the presented scanning probe lithography

**Figure 4:**
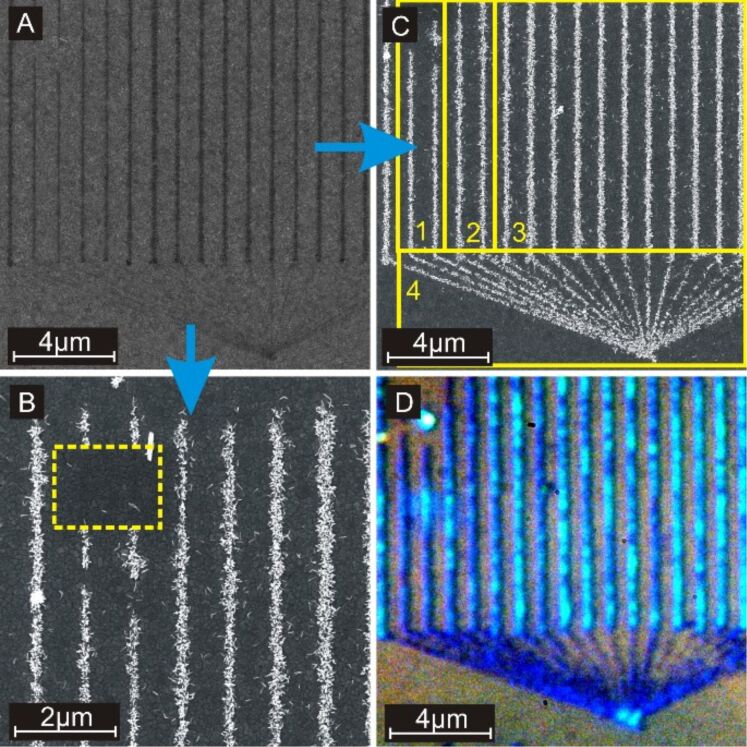
Panels A to C show SEM images. Panel A is the selectively scratched area before hydrothermal growth on an anatase layer. Panel B shows scratched lines after the hydrothermal growth process. Inside the yellow rectangle, the substrate was scratched in an identical way, but the growth was suppressed by an intensive electron-beam exposure. Panel C displays the area shown in panel A after the hydrothermal growth process. The image is separated in different areas marked by yellow rectangles and labeled by yellow numbers. The lines in area 1 are scratched 32 times per line, in area 2, 64 times per line, in area 3, 128 times per line and in area 4, only twice per line. Panel D is a bright-field image taken with an optical microscope of the same area as shown in panel C. The colors in panel D were amplified subsequently.

Several features of the demonstrated lithography technique are presented in Figure 4. Figure 4A is an SEM image of the structure after the writing process but before the hydrothermal growth of rutile nanorods.

Growth on activated seeds can be suppressed by an electron bombardment as shown in Figure 4B. Inside the yellow rectangle, the sample was scratched the same way as elsewhere but afterwards, it was exposed to an intense electron beam. The exposed area stays free of nanorods during the hydrothermal growth process. A possible explanation is surface charging by trapping of electrons from the electron beam in neutral traps such as OH groups [43]. However, this effect becomes significantly stronger with increasing beam intensity and remains for many days in a humid environment. Hence, it results more likely from electron-beam-induced surface smoothing [44–45] rather than from surface charging.

The effect of the number of repetitions on the resulting structure is shown in Figure 4C. Even a single writing step (one scratch in forward and one in backward direction) is enough to obtain a clear contrast between the treated and the pristine sample surface. More repetitions influence the obtained structure only slightly. A widening of the lines due to the degradation of the probe is not observed. It appears that the line width is almost the same at the beginning and at the end of the writing process. This is explained with the characteristics of the degradation process. As long as the tip radius is small, the applied force is concentrated on a small area and the mechanical abrasion per area is high. When the tip becomes broader the abrasion is distributed over a larger area but the applied force is still the same. This slows the degradation process down. Hence, the shape of a worn tip becomes more stable after a certain period of use. In the presented structure, the shape of the tip was almost stabilized on its path to the starting point of the line array structure. Consequently, the line width is constant for the whole sample area. Furthermore, the surface roughness of the anatase TiO_2_ film causes a dithering movement of the tip that broadens the line width. This effect disconnects the line width from the shape of the tip partly. The density of nanorods is controlled slightly with the number of repetitions. In Figure 4C, the density was increased from area 1 to 2 by roughly 20% and from area 2 to 3 by roughly 15%. This outcome is reasonable since more scratching processes generate more seed nanoparticles. A saturation of the density with an increasing number of repetitions is also expected because the density cannot increase anymore if the area of the written line is covered with nanorods completely. The average size and shape of the nanorods is given by the parameters of the growth solution and growth process and does not depend on the writing process.

Figure 4D shows that light scattering is enhanced by the TiO_2_ nanorod lines locally. This is related to the high refractive index of TiO_2_, the size of the nanorods, and the dielectric antenna effect of the stringed nanorods [46].

To test the reliability of the presented method, the writing process was repeated with several probes. The spring constant is different for each probe even if they are taken from the same fabrication batch. The value range for the spring constant given by the manufacturer is 12–103 N/m, which results in a value range for the applied force of 4–32 μN. However, the spring constants of the used probes have been usually in the lower third of the given range. It was not observed that different probes result in changes of the presented structure.

**Figure 5:**
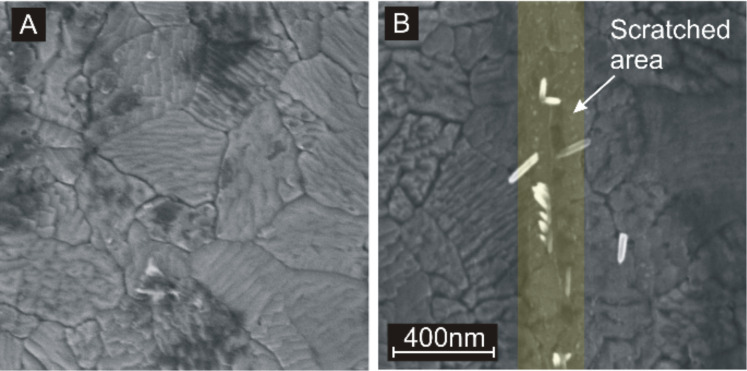
SEM images of a sample where the anatase layer is covered with a 3 nm thin SiO_2_ layer. The scratched area is shown before (panel A) and after (panel B) the hydrothermal growth process. The density of nanorods is much less compared to Figure 4. The yellow area marks roughly the region where the AFM tip scratched the sample.

Last but not least, a look at the penetration depth of this method is taken. For this purpose, a 3 nm thin SiO_2_ layer was deposited on the anatase film. Even after scratching hundreds of times at the same position with the highest employable force (8 μN), the growth was triggered only in a few places as demonstrated in Figure 5. Since significantly less force was applied during scratching the uncovered anatase film, it is reasonable that the AFM tip technique penetrates not more than the upper 1–3 nm of the anatase film. In addition, silicon nanoparticles being scraped off the AFM tip might act as nucleation sites such as other dirt particles appearing in the growth solution. However, the absence of nanorods on SiO_2_ indicates that the growth of rutile nanorods is not triggered by silicon nanoparticles.

It should be noted that the applied force during scratching is much higher than used for conventional AFM surface characterization in contact mode. Consequently, the AFM tip is worn out faster than during a standard surface characterization. Although the critical force that is needed for activating the hydrothermal growth is hard to determine, it is reasonable that the minimal required force depends on the exact kind and shape of the tip. Nanorods grown on these structures are omnidirectional. Due to the small tip radius, the resolution of this method is excellent and it is inexpensive and faster compared to electron-beam lithography and similar methods providing a position-controlled growth of semiconducting TiO_2_ nanostructures.

### Advantages and disadvantages of the scratching method

As demonstrated in Figure 2, the nanorod structures obtained with scanning probe and electron-beam lithography are equal. This report shows the proof of principle of this technique, and there are certainly many features that have to be improved for commercial applications. A need for improvement is required with respect to the rapid ageing of expensive probes, non-linearities of the scanned field, and patterning of large areas. Additionally, the writing speed of the scanning probe method is slower compared to electron-beam lithography. The presented multi-pass lines should be replaced by a single-pass writing process with higher resolution after further development. Hereby, the lifetime of the probes is extended by using commercial full-diamond probes. Furthermore, a slower degradation would preserve the resolution for large writing projects or mass production. A trivial improvement of the writing speed is achieved by special high-speed AFM devices. Simple structures such as line arrays are created much faster by employing cantilever arrays [47].

Besides these technically challenges, there are some clear advantages of this method. First, it works under ambient conditions, and the setup is inexpensive compared to electron-beam lithography. Nevertheless, the resolution is roughly of the same order of magnitude. Second, it is a direct writing method, and scratching influences only a very thin region at the sample surface and the majority of the seed film remains unchanged. As a consequence, this method can be applied to ultrathin films of a few nanometers thickness only. Third, this technique does not need any pre- and post-processing such as resist handling. This gives the opportunity to apply this method on very uneven or pre-structured substrates. Furthermore, it does not matter if the seed is a film or bulk material and by which technique the inactive seed is deposited.

## Conclusion

We demonstrated the position-controlled hydrothermal growth of rutile TiO_2_ nanorods on polycrystalline anatase TiO_2_ films using a new scanning probe lithography method. The writing with the scanning probe induces scratches in the anatase film and forms anatase nanoparticles. These nanoparticles are transformed during the hydrothermal growth process into rutile nanoparticles providing the growth of rutile nanorods locally. Hence, this direct writing method affects only the surface of the anatase films, and it can be applied to ultrathin films as well as to bulk or pre-structured samples. Other possible growth promoting effects such as surface charging and silicon dusk particles could be excluded. It was demonstrated that the activation can be erased with an intensive electron beam. Compared to other lithography methods with similar resolutions, this technique is quite inexpensive and can be applied under ambient conditions. Therefore, it complements the versatile toolkit of advanced scanning probe lithography methods [48].

## Supporting Information

File 1 Effect of the writing process on the tip.
