# Supplementary material for: Advanced scanning probe lithography using anatase-to-rutile transition to create localized TiO2 nanorods
Source: Beilstein J Nanotechnol. 2019 Feb 8;10:412–8. doi: 10.3762/bjnano.10.40 (PMC6369993; doi:10.3762/bjnano.10.40)
Supplement: File 1 — Effect of the writing process on the tip. [file Beilstein_J_Nanotechnol-10-412-s001.pdf]

BEILSTEIN JOURNAL OF NANOTECHNOLOGY

Supporting Information

for

Advanced scanning probe lithography using anatase-to-rutile transition to create localized TiO_2 nanorods

Julian Kalb, Vanessa Knittel and Lukas Schmidt-Mende

Beilstein J. Nanotechnol. **2019,** *10,* 412–418. doi:10.3762/bjnano.10.40

Effect of the writing process on the tip

License and Terms: This is a supporting information file under the terms of the Creative Commons Attribution License (http://creativecommons.org/licenses/by/4.0). Please note that the reuse, redistribution and reproduction in particular requires that the authors and source are credited.
The license is subject to the *Beilstein Journal of Nanotechnology* terms and conditions: (https://www.beilstein-journals.org/bjnano)

The effect of the writing process on the tip was investigated with SEM. Figure S1 shows the tip before the writing process and after writing 5 lines with a length of 10 μm for 256 times, respectively. The tip radius increased significantly. The worn probe is not spherical anymore and the diameter of the contact area is roughly 400 nm. However, it was not observed that the line width increased to 400 nm. Even after intensive writing processes, the average line width is less than 200 nm (Figure 2 of the manuscript) which indicates that only a minor part of the worn tip is in contact with the sample surface.

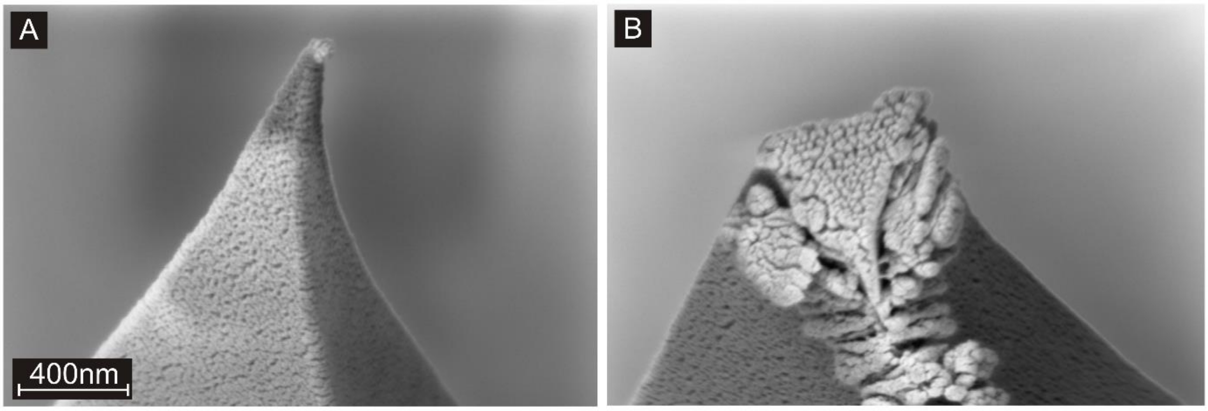

Figure S1: SEM images of a new (A) and used (B) AFM tip. The fine structure on the surface is caused by a thin gold layer which was sputtered on the tips to increase their conductivity and hence, to obtained sharper SEM images.
